# The TLR4/NF-κB/MAGI-2 signaling pathway mediates postoperative delirium

**DOI:** 10.18632/aging.203955

**Published:** 2022-03-16

**Authors:** Wei Zhang, Ruohan Wang, Jingli Yuan, Bing Li, Luyao Zhang, Yangyang Wang, Ruilou Zhu, Jiaqiang Zhang, Ting Huyan

**Affiliations:** 1 Department of Anesthesiology and Perioperative Medicine, Henan Provincial People’s Hospital, People’s Hospital of Zhengzhou University, Zhengzhou 450003, Henan Province, China; 2 Department of Anesthesiology and Perioperative Medicine, Henan University People’s Hospital, Henan Provincial People’s Hospital, Zhengzhou 450003, Henan Province, China; 3 Key Laboratory for Space Biosciences and Biotechnology, Institute of Special Environment Biophysics, School of Life Sciences, Northwestern Polytechnical University, Xi’an 710072, Shaanxi Province, China

**Keywords:** postoperative delirium, elderly patients, neuroinflammation, nuclear factor-κ-gene binding, membrane-associated guanylate kinase-2

## Abstract

Purpose: To evaluate the TLR4/NF-κB/MAGI-2 signaling pathway in postoperative delirium.

Methods: Elderly patients aged 65-80 years who received unilateral hip arthroplasty under subarachnoid anesthesia were included. Pre-anesthesia cerebrospinal fluid and perioperative blood samples were collected. After follow-up, patients were divided into two groups according to the occurrence of postoperative delirium (POD) after surgery. The potential differentially expressed proteins in the two groups were determined by proteomics assay and subsequent western blot validation. A POD model of aged mice was established, and the TLR4/NF-κB/MAGI-2 signaling pathway was determined.

Main findings: The IL-1β and TNF-α levels in pre-anesthesia cerebrospinal fluid and postoperative blood were higher in patients who developed POD than in those patients who did not. Compared with non-POD patients, MAGI-2 was highly expressed in POD patients, as validated by proteomics assays and western blotting. Higher p-NF-κB-p65, TLR4 and MAGI-2 in POD patients were detected by western blot. The POD model in aged mice was successfully established and verified by three behavioral tests. Postoperative inflammatory cytokines and the TLR4/NF-κB/MAGI-2 signaling pathway were increased in mice with POD. Inhibiting TLR4/NF-κB/MAGI-2 signaling pathway could reduce postoperative delirium.

Conclusions: The TLR4/NF-κB/MAGI-2 signaling pathway mediates POD.

## INTRODUCTION

Delirium is a cognitive disorder characterized by acute and fluctuating impairment of attention and consciousness [1]. Postoperative delirium (POD) is a serious problem and is associated with prolonged hospitalization and increased mortality. Although its incidence is 2-3% in the general surgical population, it has been reported to occur in up to 50-70% of high-risk patient populations. The incidence of postoperative delirium is much higher in patients over 60-70 years of age, reaching 10-20%. Advanced age is considered to be one of the important risk factors for POD [2]. The proportion of elderly patients undergoing anesthesia for surgery is increasing year by year, and postoperative delirium is a common complication in the elderly surgical population, with significant sequelae and associated medical burden.

Animal and human studies have led to the development of several hypotheses regarding the pathophysiology of POD, which has led to the proposal and development of new treatments. However, limited therapeutic options are currently available for clinical use. To date, pharmacotherapeutic options for delirium are limited. Presumably, current treatment options are also limited once significant delirium is present. Recent research has uncovered more about the pathophysiology of delirium, although this has not yet yielded an effective treatment. Further exploration of the mechanisms of its occurrence is necessary to reduce the incidence of delirium [3–5].

Systemic and neurological inflammatory mediators are significantly increased after surgery and remain high in the postoperative period. Increased levels of neuroinflammation are an important feature of the aging brain. Recent studies have shown that neuroinflammation is an important causal mechanism for the development of postoperative cognitive dysfunction (POCD) in elderly patients and that suppression of neuroinflammation levels may improve perioperative neurocognitive deficits [6–9]. Activation of the Toll-like receptor 4/nuclear factor-κB (TLR4/NF-κB) signaling pathway can be involved in the cellular immune response, inflammatory response, and anti-apoptosis-related gene transcription. TLR4/NF-κB activation induces proinflammatory factor (IL-1β, IL-6, TNF-α) expression, and high expression of proinflammatory factors in turn activates TLR4/NF-κB, a signaling pathway that mediates cognitive dysfunction through its downstream neuroinflammatory factors [10–12]. It has been shown that TLR4/NF-κB is involved in sevoflurane anesthesia-mediated POCD [10] and that inhibition of this signaling pathway helps to attenuate POCD in aged mice [13]. These studies all suggest that the TLR4/NF-κB signaling pathway and its mediated neuroinflammation play an important role in the development of POCD. POD is an important POCD type. Whether TLR4/NF-κB-mediated neuroinflammation is involved in the development of POD is unclear, and its specific mechanism needs to be further investigated.

The morphological and functional integrity of neuronal synapses is disrupted during the development of POCD. Inflammatory changes may disrupt the blood–brain barrier and facilitate the migration of macrophages into the brain, damaging synapses and neurons and ultimately leading to POCD [14]. Normalization of synaptic transmission favored alleviating POCD through suppression of neuroinflammation [15]. Synaptic adhesion molecules are essential for synapse formation and maintenance, with scaffolding proteins involved in synaptic processes. Scaffolding proteins are involved in the transport and aggregation of receptors and the dynamic transition of synaptic components and play an important role in synaptic stability [16]. The membrane-associated guanylate kinase (MAGUK) family includes a series of synaptic molecules that play an important role in synaptic stability and signal transduction, among which membrane-associated guanylate kinase-2 (MAGI-2) is a key synaptic scaffolding protein consisting of six PDZ domains, one guanylate kinase (GK) domain and two WW proteins. MAGI-2 is a key synaptic scaffold protein consisting of six PDZ domains, one guanylate kinase (GK) domain, and two WW domains and is mainly expressed in brain tissue [17]. Previous studies have shown that the MAGUK family is involved in learning and memory functions [18, 19]. Recently, it has been shown that the MAGI-2 differential gene in elderly patients with Alzheimer’s disease is expected to be an important screening marker as a differential gene for Alzheimer’s disease in the elderly [20], but whether MAGI-2 is involved in the development of POD is not clear.

Whether TLR4/NF-κB signaling pathway-mediated neuroinflammation leads to POD by regulating MAGI-2 expression is unclear. In this study, we screened target genes by proteomic analysis in POD patients and validated them *in vitro*, revalidated them by animal experiments, and finally found that the TLR4/NF-κB/MAGI-2 signaling pathway could mediate elderly patients with POD.

## RESULTS

**Figure 1:**
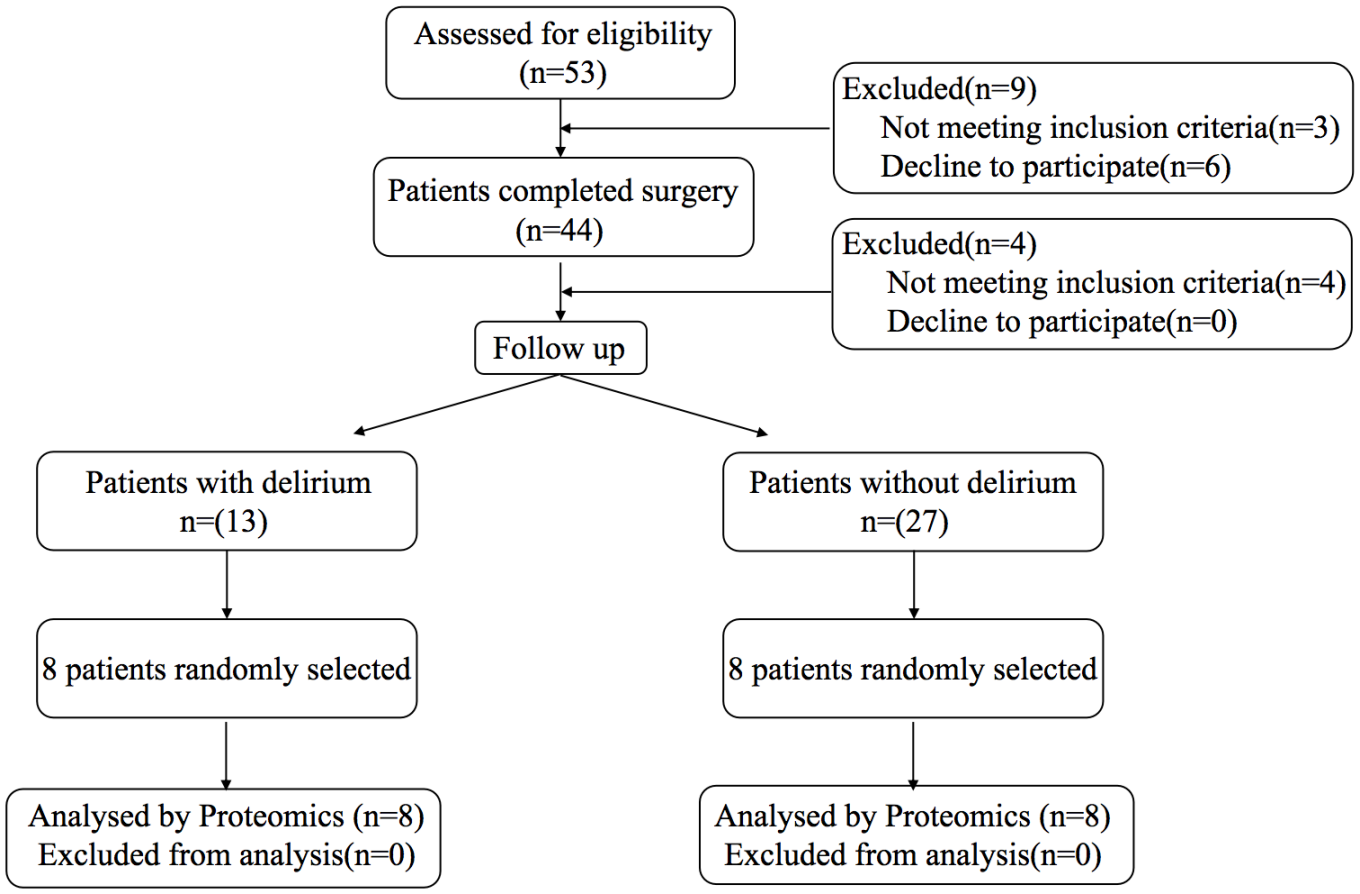
Study flow diagram for clinical trial.

**Figure 2:**
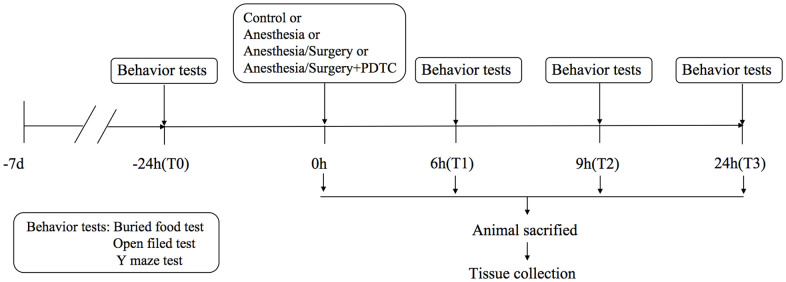
Study flow diagram for animal experiment.

The flow chart of the experiment is shown in Figure 1 (clinical trial) and Figure 2 (animal experiment).

### Comparison of patient characteristics

A total of 53 patients were initially enrolled in the study, and 40 patients completed the study with 13 patients developing POD. Peripheral blood and preoperative cerebrospinal fluid from eight patients with delirium and eight patients without delirium were selected for relevant testing and validation. The characteristics are shown in Table 1.

**Table 1:** Characteristics of the patients.

	**Patients with delirium (n=13)**	**Patients without delirium (n=27)**	***P*-value**	**Patients with delirium for proteomics (n=8)**	**Patients without delirium for proteomics (n=8)**	***P*-value**
Age(yr)	75±7	75±5	0.93	73±7	75±6	0.38
Gender (Male/Female)	11/16	4/9	0.73	3/5	5/3	0.62
ASA classification (II/III)	8/19	2/11	0.46	2/6	3/5	0.99
BMI (kg/m^2^)	24±3	25±2	0.28	23±3	25±2	0.34
Operation duration (min)	123±43	120±32	0.87	165±55	124±35	0.09
Blood loss (ml)	209±54	210±30	0.95	194±62	215±30	0.39
Urinary output (ml)	218±74	202±49	0.47	195±83	201±60	0.87
Education background			0.41			0.56
Illiterate	16	5		4	3	
Primary school	8	5		2	4	
Junior high school and above	3	3		2	1	
Smoke history (yes/no)	14/13	9/4	0.33	5/3	3/5	0.62
Diabetes type II (yes/no)	20/7	8/5	0.47	6/2	4/4	0.61
Arterial hypertension (yes/no)	17/10	5/8	0.18	5/3	7/1	0.57
Coronary artery disease (yes/no)	16/11	10/3	0.32	8/0	6/2	0.47

ASA, American Society of Anesthesiologist; BMI, Body Mass Index.

### Postoperative follow-up

**Figure 3:**
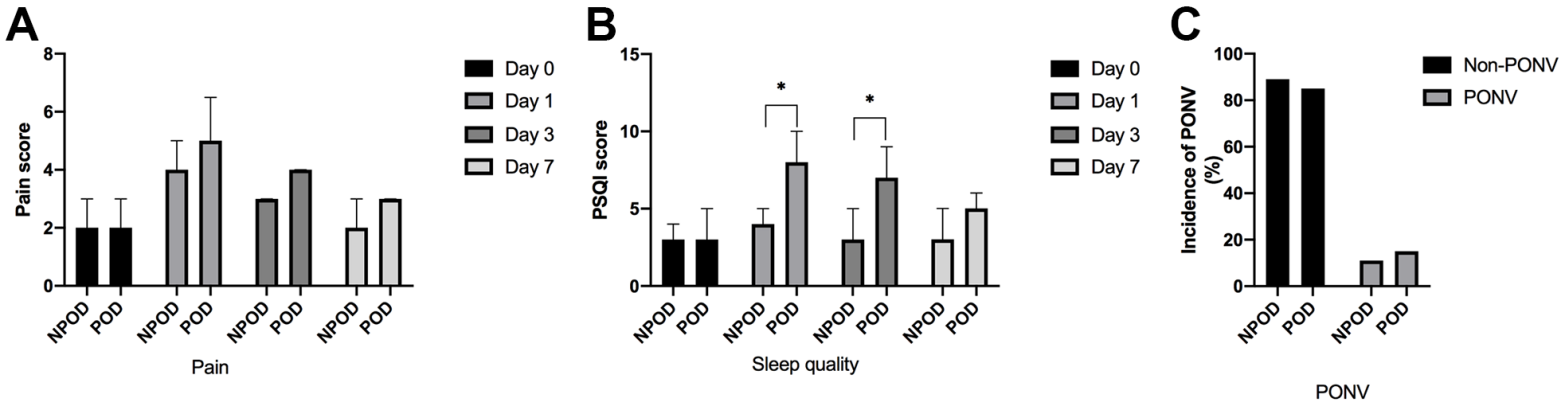
**Results of postoperative follow-up.** There were no differences in postoperative pain scores (**A**). Compared with non-POD patients, POD patients had higher PSQI scores on postoperative Day 1 and postoperative Day 3 (**B**). No differences of incidence of nausea and vomiting were found between the two groups (**C**). POD: patients with postoperative delirium; NPOD: patients without postoperative delirium; Day 0: the day before surgery; Day 1: the first day after surgery; Day 3: the third day after surgery; Day 7: the seven day after surgery or the day patient was discharge; PONV: postoperative nausea and vomiting. Compared with NPOD, ^*^p<0.05.

Postoperative follow-up was performed by a dedicated anesthesia nurse, and patients were divided into a POD group and a non-POD group according to whether they developed delirium after surgery. Compared with non-POD patients, POD patients had higher PSQI scores on postoperative Day 1 and postoperative Day 3 (Figure 3B). There were no differences in postoperative pain scores (Figure 3A) or incidence of nausea and vomiting (Figure 3C) between the two groups.

### Changes in blood and cerebrospinal fluid inflammatory indicators

**Figure 4:**
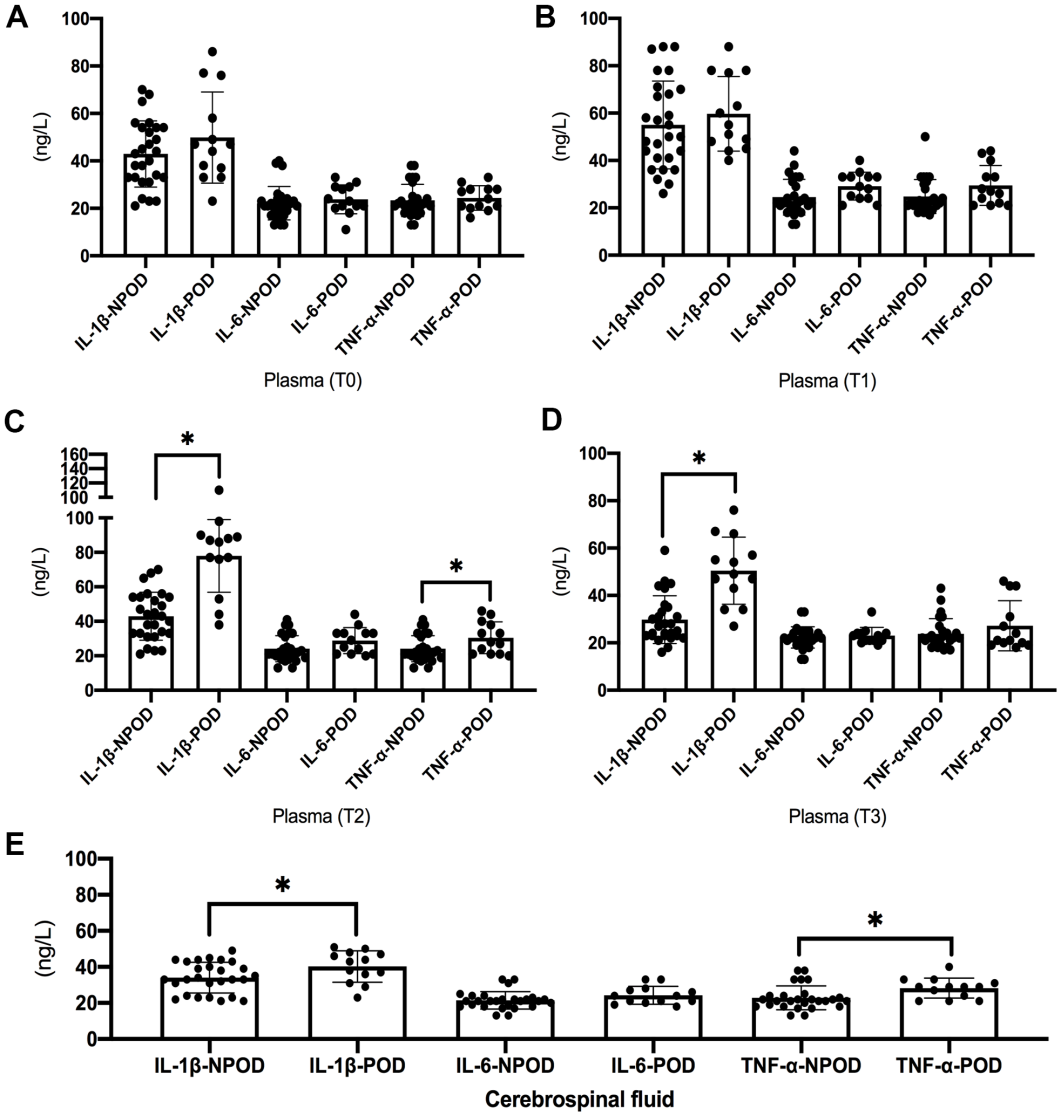
**Results of inflammatory indicators in blood and cerebrospinal fluid.** There was no statistically significant difference in the levels of IL-6, IL-1β, and TNF-α between POD and non-POD patients on preoperative and postoperative Day 1 (**A**, **B**). The levels of IL-1β and TNF-α on postoperative Day 3 were higher in POD patients than in non-POD patients (**C**). The levels of IL-1β on postoperative Day 7 were higher in POD patients than in non-POD patients (**D**). The preoperative levels of IL-1β and TNF-α in cerebrospinal fluid were higher in POD patients than in non-POD patients (**E**). POD: patients with postoperative delirium; NPOD: patients without postoperative delirium; Compared with NPOD, ^*^p<0.05.

There was no statistically significant difference in the levels of IL-6, IL-1β, and TNF-α between POD and non-POD patients on preoperative and postoperative Day 1 (Figure 4A, 4B); on postoperative Day 3, the levels of IL-1β and TNF-α were higher in POD patients than in non-POD patients (Figure 4C); on postoperative Day 7, the levels of IL-1β were higher in POD patients than in non-POD patients (Figure 4D).

The preoperative levels of IL-1β and TNF-α in cerebrospinal fluid were higher in POD patients than in non-POD patients (Figure 4E).

### Results of proteomics

**Figure 5:**
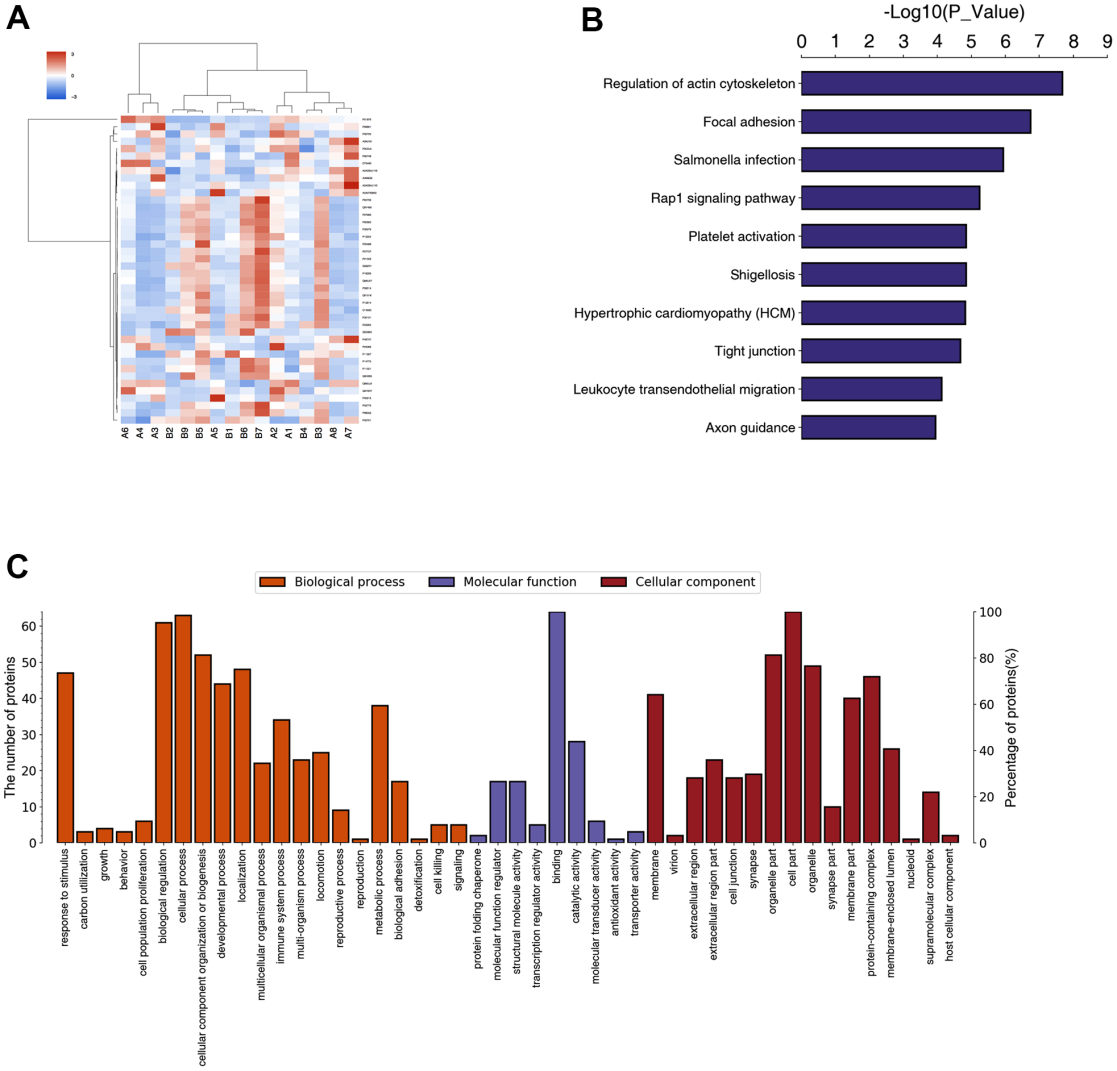
**Result of proteomics in POD patients and non-POD patients.** Eight POD patients and eight non-POD patients were randomly selected, from whom peripheral blood on the third postoperative day (T2) was collected for testing through Data Independent Acquisition (DIA) proteomics study. A total of nine proteins were upregulated and 55 proteins were downregulated in the peripheral blood of POD patients compared with non-POD patients (**A**). KEGG pathway annotation and enrichment analysis were shown in (**B**). The GO annotation and enrichment analysis were shown in (**C**).

According to the screening criteria, expression difference fold greater than 1.5-fold (up- and downregulation) and P value (t test) less than 0.05, a total of nine proteins were upregulated and 55 proteins were downregulated in the peripheral blood of POD patients compared with non-POD patients (Figure 5A). KEGG pathway annotation and enrichment analysis showed that the regulation of actin cytoskeleton, and focal adhesion were highly expressed (Figure 5B). The GO annotation and enrichment analysis were performed in three aspects: biological process, molecular function, and cellular component, and the results of biological process showed that the differential proteins were concentrated in response to stimulus, biological proliferation, cellular process, cellular component organization, or biogenesis. Molecular function results showed that the differential proteins were concentrated in binding and catalytic activity. The cellular component results showed that the differentially expressed proteins were concentrated in binding and catalytic activity. The cellular component results also showed that the differentially expressed proteins were concentrated in cell parts, organelle parts, organelles, membranes, and protein-containing complexes (Figure 5C).

### Proteomics-based validation of human peripheral blood

**Figure 6:**
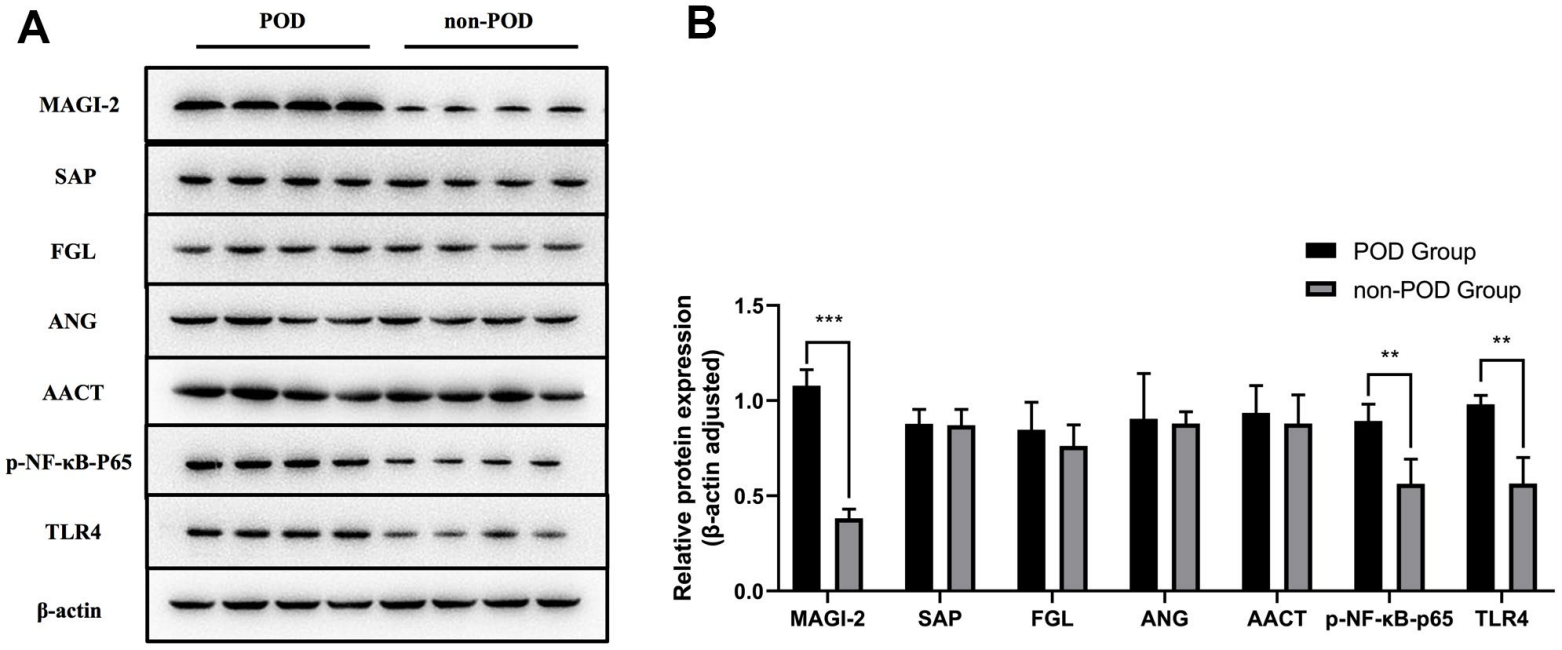
**Proteomics-based validation of human peripheral blood.** Based on the results of DIA proteomics and the hypothesis of this study, peripheral blood on the third postoperative day (T2) from eight POD patients and eight non-POD patients with DIA proteomics was validated by western blot analysis for selected differential proteins and the TLR4/NF-κB signaling pathway (**A**). The expression of MAGI-2 in the peripheral blood of POD patients was significantly higher than that of non-POD patients (**B**). TLR4/NF-κB pathway-related protein expression is shown in Figure 6B. Compared with non-POD patients, p-NF-κB-p65 and TLR4 were highly expressed in POD patients (**B**). POD: patients with postoperative delirium; non-POD: patients without postoperative delirium. Compared with non-POD group, ^*^p<0.05.

With reference to the differentially expressed proteins in proteomics and combined with the results of KEGG and GO analysis, five proteins with high expression in delirium patients were selected: MAGI-2, SAP, FGL, ANG, and AACT. After western blot validation, the expression of MAGI-2 in the peripheral blood of POD patients was significantly higher than that of non-POD patients (Figure 6A). TLR4/NF-κB pathway-related protein expression is shown in Figure 6. Compared with non-POD patients, p-NF-κB-p65 and TLR4 were highly expressed in POD patients (Figure 6B).

### Postoperative behavior test in aged mice

**Figure 7:**
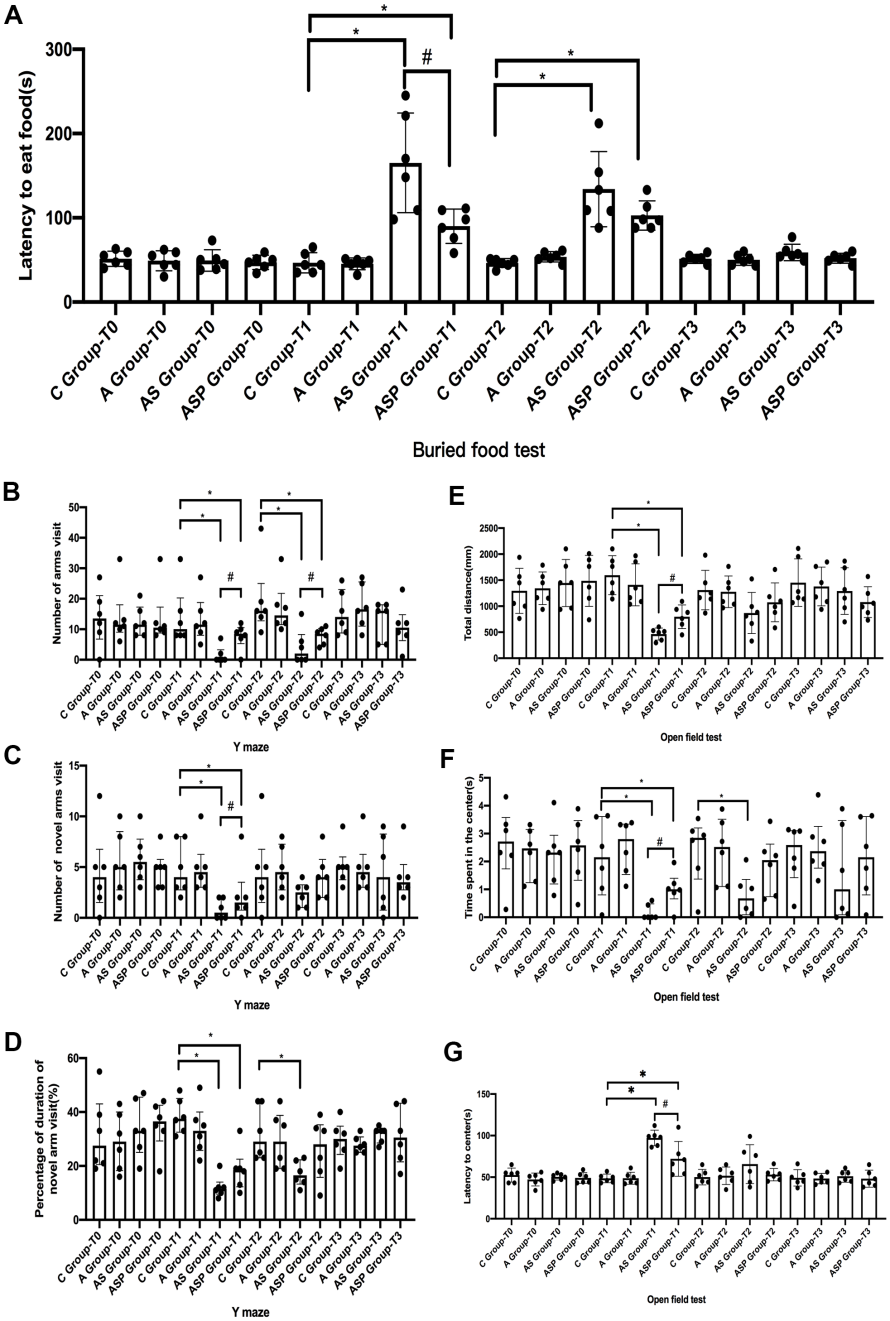
**Postoperative behavior test in aged mice.** Buried food test (**A**): the latency time to eat food was longer in AS and ASP groups than in C group at 6 h after surgery; the latency time of to eat food was shorter in ASP group at 6 h after surgery compared with AS group; and the latency time to eat food was longer in AS and ASP groups at 9 h after surgery compared with C group. Y-maze test (**B**–**D**): The total number, percentage of duration, and number of novel arm visits were decreased in the AS and ASP groups than in the C group at 6 h after surgery; the number of arms visit and number of novel arm visit were increased in the ASP group compared with the AS group. Compared with the C group, the number of arms visits at 9 h postoperatively was decreased in the AS and ASP groups, and the percentage of duration of novel arm visits was lower in the AS group than in the C group; the number of arms visits at 9 h postoperatively was higher in the ASP group than in the AS group. Open field test (**E**–**G**): the total distance and time spent in the center were shorter in the AS and ASP groups than those in C group and the latency to center was longer than those in C group at 6 h postoperatively. Compared with the AS group, the total distance and time spent in the center in the ASP group were increased and the latency to center was decreased at 6 h postoperatively. Compared with the C group, the time spent in the center was shorter in the AS group at 9 h postoperatively. Compared with the C group, ^*^p<0.05; compared with the AS group, ^#^*p*<0.05.

Buried food test: There was no difference between groups in the latency time of to eat food in all mice at 24 h before surgery, and the latency time to eat food was longer in AS and ASP groups than in C group at 6 h after surgery; the latency time of to eat food was shorter in ASP group at 6 h after surgery compared with AS group; and the latency time to eat food was longer in AS and ASP groups at 9 h after surgery compared with C group (Figure 7A).

Y-maze test: There was no difference in the total number of arms visit, the percentage of duration of novel arm visit, and the number of novel arm visit for all mice before surgery. The total number, percentage of duration, and number of novel arm visits were decreased in the AS and ASP groups than in the C group at 6 h after surgery; the number of arms visit and number of novel arm visit were increased in the ASP group compared with the AS group. Compared with the C group, the number of arms visits at 9 h postoperatively was decreased in the AS and ASP groups, and the percentage of duration of novel arm visits was lower in the AS group than in the C group; the number of arms visits at 9 h postoperatively was higher in the ASP group than in the AS group (Figure 7B–7D).

Open field test: The total distance, time spent in the center and latency to center did not differ between all mice before surgery. The total distance and time spent in the center were shorter in the AS and ASP groups than those in C group and the latency to center was longer than those in C group at 6 h postoperatively. Compared with the AS group, the total distance and time spent in the center in the ASP group were increased and the latency to center was decreased at 6 h postoperatively. Compared with the C group, the time spent in the center was shorter in the AS group at 9 h postoperatively (Figure 7E–7G).

### Results of hippocampal proteins in aged mice

**Figure 8:**
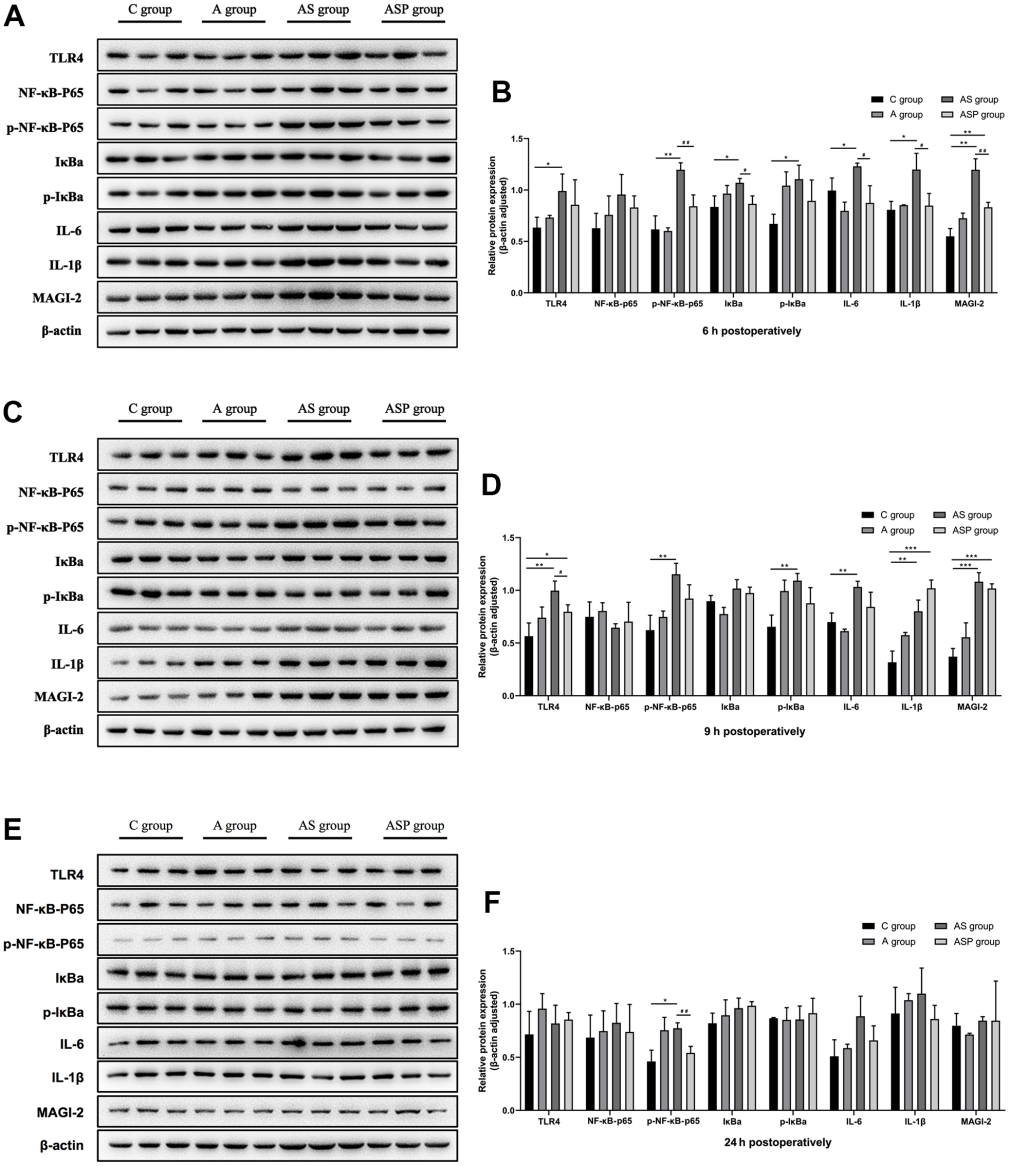
**Expression of hippocampal proteins of POD model in aged mice.** Six hour postoperatively (**A**, **B**) Compared with the C group, TLR4, p-NF-κB p65, IκBα, p-IκBα, IL-6, IL-1β, and MAGI-2 expression was elevated in the AS group. MAGI-2 expression was higher in the ASP group than in the C group. p-NF-κB p65, IκBα, IL-6, IL-1β and MAGI-2 expression was lower in the ASP group than in the AS group. Compared with the C group, ^*^p<0.05, ^**^p<0.01, ^***^p<0.001; compared with the AS group, ^#^*p*<0.05, ^##^*p*<0.01. Nine hour postoperatively (**C**, **D**) Compared with the C group, the expression of TLR4, p-NF-κB p65, p-IκBα, IL-6, IL-1β, and MAGI-2 was elevated in the AS group, and the expression of TLR4, IL-1β and MAGI-2 in the ASP group was higher than that in the C group; compared with the AS group, the expression of TLR4 in the ASP group was decreased (Figure 8). Compared with the C group, ^*^p<0.05, ^**^p<0.01, ^***^p<0.001; compared with the AS group, ^#^*p*<0.05, ^##^*p*<0.01. Twenty-four hour postoperatively (**E**, **F**) Compared with the C group, p-NF-κB p65 expression was elevated in the AS group, and the p-NF-κB p65 expression was lower in the ASP group than in the AS group. Compared with the C group, ^*^p<0.05, ^**^p<0.01, ^***^p<0.001; compared with the AS group, ^#^*p*<0.05, ^##^*p*<0.01.

Six hour postoperatively (Figure 8A, 8B) Compared with the C group, TLR4, p-NF-κB p65, IκBα, p-IκBα, IL-6, IL-1β, and MAGI-2 expression was elevated in the AS group. MAGI-2 expression was higher in the ASP group than in the C group. p-NF-κB p65, IκBα, IL-6, IL-1β and MAGI-2 expression was lower in the ASP group than in the AS group.

Nine hour postoperatively (Figure 8C, 8D) Compared with the C group, the expression of TLR4, p-NF-κB p65, p-IκBα, IL-6, IL-1β, and MAGI-2 was elevated in the AS group, and the expression of TLR4, IL-1β and MAGI-2 in the ASP group was higher than that in the C group; compared with the AS group, the expression of TLR4 in the ASP group was decreased (Figure 8).

Twenty-four hour postoperatively (Figure 8E, 8F) Compared with the C group, p-NF-κB p65 expression was elevated in the AS group, and the p-NF-κB p65 expression was lower in the ASP group than in the AS group.

## DISCUSSION

To the best of our knowledge, this is the first study to elucidate the effect of the TLR4/NF-κB/MAGI-2 signaling pathway on POD through clinical trials and animal studies. Generally, our study was divided into two parts. The clinical trial part was conducted in elderly orthopedic patients and found that the preoperative cerebrospinal fluid inflammation levels and postoperative peripheral blood inflammation levels were significantly higher in patients with POD than in those without POD. By using proteomic techniques, it was found that MAGI-2 in peripheral blood was highly expressed in POD patients, and further validation was performed by immunoblotting to find that the TLR4/NF-κB signaling pathway and MAGI-2 were more highly expressed in POD patients. To further validate the results of the human study, we established a POD model in aged mice and found that the mice showed behavioral impairment after surgery, along with high expression of the TLR4/NF-κB signaling pathway and MAGI-2 in their hippocampal tissues. After inhibiting NF-κB expression, the behavioral impairment of the mice after surgery was improved, while their MAGI-2 expression was downregulated, further validating the results of the human study.

The incidence of postoperative sleep disturbances is high, especially in elderly patients [21]. Postoperative sleep disorders are thought to be closely related to postoperative delirium, and patients with postoperative sleep disorders are more likely to develop postoperative delirium [22, 23]. The results of this study showed that patients who developed POD had poorer sleep quality on postoperative Day 1 and postoperative Day 3 than non-POD patients, suggesting that the development of postoperative delirium is closely related to postoperative sleep disorders, but whether there is a causal relationship between the two is unclear. It has also been shown that sleep disorders can lead to increased levels of postoperative neuroinflammation [24], which is consistent with the increased levels of postoperative peripheral inflammation in patients with delirium in the present study.

Hip arthroplasty patients are prone to postoperative delirium after surgery, especially elderly patients. It has been shown that inflammatory factor levels in the cerebrospinal fluid of elderly patients are closely associated with postoperative delirium and that higher levels of preoperative cerebrospinal fluid inflammation are associated with a greater risk of postoperative delirium. Previous studies have shown that preoperative peripheral injuries, such as fractures, are associated with increased inflammatory mediators in the CSF [25, 26]. Previous studies have shown that peripheral blood IL-6 is associated with cognitive function in elderly patients after knee surgery [27] and that peripheral inflammatory levels of inflammatory factors, represented by IL-6, are associated with neurological dysfunction, along with levels of inflammation in the central nervous system [28]. The results of this study showed that the levels of preoperative cerebrospinal fluid IL-1β and TNF-α were higher in delirium than in non-delirium patients, suggesting that patients with high levels of preoperative neuroinflammation are prone to delirium postoperatively.

The time point of postoperative delirium is concentrated in the immediate postoperative period to 3 days postoperatively and generally recovers within 1 week postoperatively [8, 29, 30]. In this study, patients were evaluated by dedicated personnel from the immediate postoperative period to 1 week postoperatively (or when patients were discharged from the hospital), and this follow-up time basically covered the high incidence of postoperative delirium. It is difficult and unethical to collect cerebrospinal fluid from postoperative patients; therefore, peripheral blood was collected from postoperative patients for measurement. It has been shown that the level of inflammation in peripheral blood is closely related to postoperative delirium, and patients with a high level of inflammation are prone to delirium. It has been reported that postoperative elevation of peripheral C-reactive protein (CRP) and interleukin 6 concentrations is associated with higher risks of postoperative delirium [31]. The results of this study showed that there were dynamic changes in postoperative peripheral blood inflammation levels. There was no difference in the levels of IL-6, IL-1β, and TNF-α between POD and non-POD patients on the first postoperative day, but by the third postoperative day, the levels of IL-1β and TNF-α were higher in POD patients than in non-POD patients, and the levels of IL-1β were still higher in POD patients than in non-POD patients on the seventh postoperative day. The results of this study showed that postoperative inflammation levels were higher in delirium patients than in non-delirium patients, and combined with the results of cerebrospinal fluid, we concluded that high preoperative neuroinflammation and high postoperative inflammation levels were closely related to postoperative delirium.

To further explore the mechanism of postoperative delirium and screen for valuable signaling molecules, we screened using proteomic techniques and verified by subsequent experimental techniques that high expression of MAGI-2 in peripheral blood was an important marker of postoperative delirium, and the level of MAGI-2 in peripheral blood was significantly higher in POD patients than in non-POD patients. The results of the present study suggest that both inflammation levels and MAGI-2 are higher in delirium patients than in non-delirium patients, and previous studies have shown that higher inflammation levels can in turn disrupt synaptic plasticity [32]. Previous studies have shown that representative neuroinflammation is associated with postoperative cognitive impairment, and the clinical study in this study showed that postoperative peripheral blood MAGI-2 and TLR4/NF-κB signaling pathway were higher in delirium than in non-delirium patients, and animal experiment in this study further revealed the relationship between the TLR4/NF-κB signaling pathway and MAGI-2.

Previous studies have shown that abdominal exploratory surgery under general anesthesia is considered to establish an animal model of postoperative delirium. Our animal study showed that learning ability and exploration ability were decreased, indicating that our animal model was successfully established.

The present study showed that no significant behavioral changes occurred in mice in Group A compared with C. This indicates that mice simply receiving anesthesia without surgical operation do not lead to behavioral changes, which is consistent with the findings of previous studies. Mice receiving both anesthesia and surgery experienced behavioral impairment and gradually returned to normal baseline at 24 hours postoperatively. At the same time, the expression of the TLR4/NF-κB signaling pathway and MAGI-2 in the hippocampal tissue of mice was significantly increased, which indicated that neuroinflammation represented by TLR4/NF-κB and high expression of MAGI-2 molecules were involved in the occurrence of POD. The high expression of MAGI-2 in the hippocampus of POD mice is consistent with the high expression of MAGI-2 in peripheral blood found in our clinical study, which further suggests that high expression of MAGI-2 is involved in the development of POD. To verify whether TLR4/NF-κB affects the MAGI-2 molecule and its regulatory role in POD, an NF-κB antagonist was applied before and after surgery, and the results showed that the application of an NF-κB antagonist partially ameliorated behavioral impairment in mice and downregulated TLR4/NF-κB in the hippocampus. The application of an NF-κB antagonist was followed by downregulation of hippocampal MAGI-2 expression, which further suggests that TLR4/NF-κB regulates the expression of MAGI-2 and thus promotes POD.

There are several limitations in this study. First, due to restrictions and ethical requirements, clinical samples of postoperative cerebrospinal fluid from delirium patients could not be obtained. MAGI-2, a novel marker found in our study, was found by using proteomic analysis in peripheral blood and further validated by immunoblotting. A subsequent animal experiment verified the results of a human study. Second, specific antagonists of MAGI-2 could not be obtained directly, so the role of the TLR4/NF-κB/MAGI-2 signaling pathway could not be further verified by directly up- or downregulating the expression of MAGI-2. The role of this signaling pathway in POD was validated more by monitoring the pattern of changes in MAGI-2 through regulation of NF-κB expression and the corresponding behavioral results. Third, in clinical studies, we further validated human peripheral blood for proteomic findings, but only those highly expressed neuroinflammation and related molecules were selected. Some of those upregulated proteins in POD patients were not verified. Finally, the intervention conditions and anesthesia methods of the clinical trial and animal trial in this study were different. However, high neuroinflammation is believed to be the common mechanism of POD between human and animal. The results in animal POD model were consistent with clinical trial in this study.

In conclusion, our study suggests that the TLR4/NF-κB/MAGI-2 signaling pathway mediates POD. Inhibition of NF-κB contribute to alleviate the POD. MAGI-2 is thought to be a novel biological marker of POD. Highly expressed MAGI-2 in peripheral blood and hippocampus is related to POD. Future research could focus on the key effect of MAGI-2 on POD.

## MATERIALS AND METHODS

This was a single-center prospective observational study conducted at Henan Provincial People’s Hospital, and the clinical study was approved by the Medical Ethics Committee of Henan Provincial People’s Hospital [2019, Lun Shen, No. 103]. The trial registry number is ChiCTR1900027276 and was registered on 07 Nov 2019 (https://www.chictr.org.cn/showproj.aspx?proj=44786). Written informed consent and information release approvals were obtained from all patients prior to their participation in the study. The study protocol complied with the 1975 Declaration of Helsinki.

### Clinical study section

Inclusion criteria: Elderly patients aged 65-80 years, ASA II-III, BMI 18-30 kg/m^2^, and who received unilateral hip arthroplasty under subarachnoid anesthesia were included.

Exclusion criteria: No history of anesthetics and/or surgery within the last 3 months, no autoimmune disease, and no history of hormone application. Patients who need this operation due to trauma; presence of contraindications to spinal anesthesia, such as bleeding and coagulation dysfunction or puncture site infection; previous history of cerebrovascular accident; Pittsburgh Sleep Quality Index Scale (PSQI) score >7; use of psychotropic drugs; bilateral hip arthroplasty; patients with preoperative delirium; refusal to participate in this trial.

Rejection criteria: Failure in subarachnoid anesthesia; those with a change in anesthesia or surgical procedure; postoperative transfer to ICU.

### Anesthesia protocol

Routine monitoring (blood pressure, pulse oximetry, ECG monitoring) was performed after the patient was admitted to the operation room, and no preoperative medication was given before anesthesia. After peripheral venous access was established and 2 ml extraction of peripheral blood was performed, the patient was placed in a lateral position, and subarachnoid puncture was performed at the L3-4 interval. One milliliter of cerebrospinal fluid was extracted with a sterile syringe after smooth cerebrospinal fluid reflux, and then 2 ml of 0.8% ropivacaine was injected into the subarachnoid space. After successful anesthesia, unilateral hip arthroplasty was performed. All patients were treated with a postoperative patient-intravenous self-controlled analgesia protocol with a pumping regimen of 1 μg/kg oxycodone and 10 mg tropisetron. All patients received intravenous parecoxib sodium injection twice a day. Patients were given intravenous oxycodone 2-4 mg when visual analog scale was larger than 4.

### Postoperative follow-up

Follow-up was performed by a full-time anesthesia nurse, and the cognitive status of patients was assessed by using the CAM scale. The follow-up time points were the day before surgery and postoperative Days 1-7 (or up to the time of patient discharge). According to the results of the CAM scale, the patients were divided into two groups: patients with or without delirium.

Pain was assessed on the day before surgery and on postoperative Days 1, 3, and 7 by using a visual analog scale; sleep quality was assessed on the day before surgery and on postoperative Days 1, 3, and 7 by the Pittsburgh Sleep Quality Index scale (PSQI); and the occurrence of postoperative nausea and vomiting was recorded.

### Sample acquisition and testing

Ten milliliters of peripheral blood were drawn from the patient at the following time points: immediately before anesthesia (T0), the first postoperative day (T1), the third postoperative day (T2), and the seventh postoperative day (or when the patient was discharged) (T3). Blood samples were placed in a heparin anticoagulation tube and centrifuged at 4° C. Finally, plasma was obtained and stored at -80° C for examination. The cerebrospinal fluid obtained intraoperatively was placed in a frozen storage tube and centrifuged at 4° C, and the supernatant was stored at -80° C for storage. The plasma and cerebrospinal fluid supernatants were assayed for IL-6, IL-1β, and TNF-α using ELISA kits (Elabscience Biotechnology Co., Ltd, China).

### Proteomics assay

Patients were divided into a POD group and a postoperative non-delirium (non-POD) group according to the postoperative CAM scale follow-up. Eight POD patients and eight non-POD patients were randomly selected by using random number table, from whom peripheral blood on the third postoperative day (T2) was collected for testing through Data Independent Acquisition (DIA) proteomics study.

The DIA proteomics assay was performed with reference to the literature [33, 34]. Proteins that met the screening criteria of differential expression ploidy greater than 1.5-fold (up- and downregulated) and P value (t test) less than 0.05 were considered differentially expressed proteins. Significant difference analysis, Gene Ontology (GO) annotation and enrichment analysis, Kyoto Encyclopedia of Genes and Genomes (KEGG) pathway annotation and enrichment analysis, and cluster analysis were used for biological information analysis.

### *In vitro* validation in human peripheral blood

Based on the results of DIA proteomics and the hypothesis of this study, peripheral blood used for DIA proteomics was validated by western blot analysis for selected differential proteins and the TLR4/NF-κB signaling pathway (n=4 in each group). After the peripheral blood was centrifuged and the supernatant was discarded, RIPA lysis buffer was added at a volume of 1:2 and lysed on ice for 30 minutes. The supernatant was collected after centrifugation at 4° C and 12,000 x g for 30 minutes. Protein quantitation of the lysates was carried out by the BCA kit (cat# PC0021, Solarbio). The lysates were separated using 12% SDS-polyacrylamide gel electrophoresis and transferred onto PVDF membranes. The blots of phosphorylated proteins were blocked with 5% BSA at room temperature for 1 h, and the blots of other proteins were blocked with 5% nonfat dry milk for 1 h. Then, the blots were incubated overnight at 4° C with the corresponding primary antibodies at the recommended concentration of the reagent specification. After washing the membrane with TBST, the corresponding HPR-labeled IgG was added, the membrane was washed after incubation, the target band was detected by ECL chemiluminescence, and pictures were observed, analyzed, and taken on the gel imaging system. The ratio of the gray value of the target protein to the internal reference protein was used to calculate the corresponding protein expression.

### Animal experiments section

### Animal selection and grouping

SPF-grade healthy male C57BL/6J mice, aged 14-16 months old, purchased from Beijing Viton Lever Co., were housed in a 12-h light/12-h dark environment (room temperature 25° C) with free access to food and water for 1 week.

The mice were randomly divided into four groups (n=6 in each group) using a random number table: control group (Group C), anesthesia-only group (Group A), anesthesia-surgery group (group AS), and AS+PDTC group (group ASP). Mice in Group C were placed in an induction box containing desiccant and carbon dioxide adsorbent to inhale 100% oxygen and did not receive anesthesia and surgical treatment; mice in Group A received 1.4% isoflurane (Lot number: 20010601, Reward Life Technology Co.Ltd., China) (100% oxygen) anesthesia treatment for 2 h; mice in group AS underwent surgery under 1.4% isoflurane (100% oxygen) anesthesia; mice in ASP group were intraperitoneally injected with 50 mg/kg PDTC (Lot number: BCCF3413, Sigma-Aldrich, USA) 30 min before and 2 h after surgery, and other protocols were the same as those in AS group.

### Establishment of the POD model

According to a previous reference [35–37], laparotomy was performed under 1.4% isoflurane (100% oxygen) anesthesia with a thermostatic heating pad to maintain body temperature and an anesthetic gas monitor to measure the concentration of isoflurane in the induction box in real time. Local anesthesia with 1% lidocaine was administered to the surgical site prior to skin incision to aid in analgesia. Before surgery, the mice were prepared with skin in the surgical area, and the surgical field and surrounding skin were disinfected with 1% vital iodine three times. Sterile surgical sheets were laid, and the skin, abdominal muscle, and peritoneum were incised layer by layer along 0.5 cm above the pubic symphysis to the subxiphoid process. The abdominal organs were explored clockwise with sterile cotton swabs for 5 min. Then, the incision was closed layer by layer with 5-0 absorbable thread, and lidocaine cream was applied to the incision for postoperative analgesia once/8 h after surgery. After surgery, mice were placed in an induction cassette containing 1.4% isoflurane mixed with 100% oxygen and maintained in anesthesia until 2 h. At the end of anesthesia, the mice were placed back into the feeding cage.

### Behavioral tests

Tests were performed at 24 h preoperatively and at 6 h, 9 h, and 24 h postoperatively. Six mice were randomly selected from each group at each time point for behavioral testing according to the sequence of the buried food test, the open field test, and the Y-maze. The field was wiped with alcohol after each experiment and dried, and the experimenter changed gloves before each test to avoid the behavioral traces of that mouse and the odor produced to affect the behavioral performance of other mice.

### Buried food test

The test mice were given two tablets/d of sweet cereal 2 d before testing. One hour before the test, the cage with clean bedding was placed in a quiet room to adapt to the test environment. The cereal was buried 0.5 cm below the bedding to prevent the mice from seeing the cereal directly, and then the mice were placed in the middle of the cage. The latency to eat food (the time from entering the cage to finding and eating the cereal) was recorded. The observation time was 5 min. If the mice did not find the cereal within 5 min, the incubation period was recorded as 300 s.

### Open field test

The mice were placed in the center of the open field and allowed to move freely. The trajectory was collected for 5 min, and the total distance traveled, time spent in the center, and latency to enter the center within 5 min were recorded using a video monitoring and analysis system (SMARTSUPER 3.0, Shenzhen Reward Life Technology Co., Ltd., China).

### Y-maze experiment

The three arms were randomly defined as: the starting arm, i.e., the arm where the mice started to enter the maze, which was always open during the experiment; the other arms, which were always open during the experiment; and the neoisolation arm, i.e., which was isolated during the training phase and opened during the testing phase. The experiment was divided into two phases: training and testing phases. In the training phase, mice were put in from the starting arm and were free to explore other areas except the neoisolation arm; after 10 min, the training ended, and the mice were put back into the rearing cage. After 2 h, the mice were put into the test phase, and they were free to explore the three arms for 5 min. Animal behavior analysis software was used to track the mice’s activities, and the number of arms visits, the percentage of duration of novel arm visits, and the number of novel arm visits were recorded.

### Specimen acquisition and testing

After the behavioral tests were completed, the intact brain tissue (n=3 in each group) was quickly removed under anesthesia with intraperitoneal injection of sodium pentobarbital (50 mg/kg), and the bilateral hippocampal tissue was quickly isolated on ice and placed at -80° C for examination.

Hippocampal tissues were lysed with RIPA lysis solution, and whole proteins were extracted, quantified by BCA, electrophoresed, and electrotransferred to PVDF membranes. The membrane was blocked with 5% nonfat dry milk for 1 h at room temperature, followed by the addition of primary antibody incubated overnight at 4° C. The membrane was washed three times with TBST for 10 minutes each time and incubated with HRP-labeled secondary antibody for 1 hour at room temperature. Similarly, the blots were also washed three times with TBST for 10 minutes each time and developed on film by the darkroom ECL method. The films were scanned, and the target protein expression was quantified by ImageJ software using β-actin (cmctag, catalog number: AT0001) as a reference.

### Statistical analysis

The statistical analysis was performed using SPSS software version 21.0 (IBM Corp., Armonk, NY, USA). Depending on data distribution, the continuous variables are recorded as mean ± SD or median (interquartile range (IQR)). Two independent sample t-tests were used to compare the continuous variables with normal distribution. To compare non-normal continuous variables, Kruskal-Wallis test was used. Frequency and percentage were recorded as categorical variables, which was analyzed using Chi-square test or Fisher exact test. *p*< 0.05 was regarded as statistically significant.
